# Obesity and Metabolic Syndrome Predict Polyneuropathy Over 5 Years in Recent‐Onset Type 2 Diabetes and Normal Glucose Tolerance

**DOI:** 10.1002/dmrr.70147

**Published:** 2026-03-03

**Authors:** Gundega Sipola, Alexander Strom, Robert Wagner, Kálmán B. Bódis, Dan Ziegler, Michael Roden, Gidon J. Bönhof, for the GDS Group

**Affiliations:** ^1^ Institute for Clinical Diabetology German Diabetes Center Leibniz Center for Diabetes Research at Heinrich Heine University Düsseldorf Düsseldorf Germany; ^2^ German Center for Diabetes Research Partner Düsseldorf Münich‐Neuherberg Germany; ^3^ Department of Endocrinology and Diabetology Medical Faculty and University Hospital Düsseldorf Heinrich Heine University Düsseldorf Düsseldorf Germany

**Keywords:** metabolic syndrome, neuropathy, obesity, overweight, polyneuropathy, type 2 diabetes

## Abstract

**Aims:**

To evaluate the impact of obesity and metabolic syndrome (MetS) on distal sensorimotor polyneuropathy (DSPN) over 5 years of recent‐onset type 2 diabetes and normal glucose tolerance (NGT).

**Materials and Methods:**

Individuals with recent‐onset type 2 diabetes and NGT (*n* = 355/181) matched for age and sex underwent reference tests of DSPN. Intraepidermal nerve fibre density (IENFD) was assessed in a subset (*n* = 100/117). Subgroups (*n* = 198/64) were reevaluated 5 years later. MetS was defined by International Diabetes Federation criteria and DSPN by the Toronto consensus criteria.

**Results:**

After adjustment for age, sex, height, smoking history, and HbA1c, IENFD was inversely associated with weight and the number of MetS components in NGT, but not in type 2 diabetes. Lower sural sensory nerve action potential was associated with higher weight in both groups. Higher baseline weight was associated with incident DSPN in NGT. Obesity and MetS at baseline were associated with higher odds of neuropathic deficits at follow‐up in type 2 diabetes (OR [95% CI] for obesity 3.00 [1.06–8.51] and MetS 8.25 [1.02–66.91]).

**Conclusions:**

Higher weight is independently associated with early nerve fibre loss and dysfunction, regardless of hyperglycaemia. In recent‐onset type 2 diabetes, obesity and MetS predict neuropathic deficits 5 years later, suggesting that timely weight management may be critical to prevent DSPN.

## 1 Introduction

Although distal sensorimotor polyneuropathy (DSPN) constitutes one of the most common and clinically challenging chronic complications of diabetes, its underlying pathogenic mechanisms and risk factors remain incompletely elucidated [1]. Current evidence suggests that DSPN develops during the early course of diabetes [2, 3] and even in prediabetes [4]. Moreover, recent findings suggest a role of obesity and metabolic syndrome in the development of DSPN [5, 6, 7].

While hyperglycaemia is considered the primary risk factor for DSPN [5], there is currently no convincing evidence from large, well‐designed studies that rigorous glycaemic control can effectively prevent or slow down the progression of DSPN in individuals with type 2 diabetes [8, 9]. Therefore, a broader approach addressing additional risk factors, particularly the cardiovascular ones, may be necessary to prevent the development of DSPN [5]. Besides hyperglycaemia, obesity is emerging as a modifiable risk factor for DSPN in both people with and without diabetes [5, 7]. With the current global prevalence of overweight and obesity estimated at 45.1% and expected to reach nearly 60% of the adult population by 2050 [10], an alarming increase in DSPN cases can be expected to be potentially attributable to obesity and obesity‐related comorbidities. In addition, some observational studies have suggested that the combination of obesity and cardiometabolic obesity‐related risk factors, collectively referred to as metabolic syndrome, is associated with polyneuropathy, irrespective of the presence of diabetes [6, 7]. However, the role of obesity, metabolic syndrome, and its individual components on small and large nerve fibre dysfunction and structural impairment in people with recent‐onset type 2 diabetes remains unclear. Furthermore, there is a lack of knowledge about the influence of obesity and other metabolic syndrome components on nerve function and morphology in persons with normal glucose tolerance (NGT), since most previous studies included a relevant proportion of participants with prediabetes [11, 12, 13, 14, 15] and/or their focus was primarily on severe obesity [16, 17].

This study aimed to assess the role and predictive value of weight, metabolic syndrome and its individual components on measures of DSPN over the first 5 years of well‐controlled type 2 diabetes compared with individuals with NGT using a comprehensive assessment of small and large nerve function and structure. We hypothesised that overweight and metabolic syndrome are associated with nerve fibre impairment and that both are predictive of declining nerve function, independent of diabetes.

## 2 Materials and Methods

### 2.1 Study Participants

Individuals were recruited from the German Diabetes Study (GDS; ClinicalTrials.gov identifier: NCT01055093) in Düsseldorf, Germany. The GDS is an ongoing, prospective study observing the natural course of diabetes and its complications [18]. The study design and profile of the Düsseldorf cohort have been previously described in detail [18]. The GDS study was approved by the Ethics Committee of Heinrich Heine University, Düsseldorf, Germany and conducted in accordance with the Declaration of Helsinki (7th revision, 2013). Written informed consent was obtained from all participants prior to enrolment.

Three hundred fifty‐five individuals with recently‐diagnosed type 2 diabetes (known diabetes duration ≤ 12 months) and 181 individuals with NGT matched for age and sex without non‐metabolic causes of peripheral neuropathy were included in the baseline cohort for cross‐sectional analysis. In the baseline cohort, intraepidermal nerve fibre density (IENFD) was quantified in 101 individuals with type 2 diabetes and 117 with NGT. The prospective analysis included 198 participants with type 2 diabetes and 64 with NGT who underwent a 5‐year follow‐up. Of these, 67 individuals with type 2 diabetes and 48 with NGT had IENFD assessment performed at baseline and 57 and 38, respectively, at follow‐up.

### 2.2 Polyneuropathy Assessment

#### 2.2.1 Electrophysiological and Quantitative Sensory Testing

Peripheral nerve function, including electrophysiological [motor nerve conduction velocity (MNCV) in the ulnar, median, and peroneal nerves, sensory nerve conduction velocity (SNCV) in the ulnar, median, and sural nerves, and sensory nerve action potential (SNAP) in the sural nerve] and quantitative sensory testing [QST; vibration perception thresholds (VPTs) at the second metacarpal bone and medial malleolus, warmth and cold detection thresholds (WDTs and CDTs, respectively) at the thenar eminence and dorsum of the foot], and clinical neuropathy scores were assessed as previously described [2, 19]. Neuropathic deficits were assessed with the Neuropathy Disability Score (NDS; abnormal ≥ 3 points), while neuropathic symptoms were evaluated with the Neuropathy Symptom Score (NSS; abnormal ≥ 3 points) [20]. DSPN was defined following the consensus of the Toronto Diabetic Neuropathy Expert Group, including subclinical, possible, probable, and confirmed stages [21].

#### 2.2.2 Skin Biopsy

Skin innervation was assessed by determining IENFD (intraepidermal nerve fibres/mm epidermis) in 50‐μm skin biopsy sections using the free‐floating method from a three‐mm skin punch biopsy specimen obtained from the distal lateral calf 10 cm proximal to the lateral malleolus under local anaesthesia as previously described [2, 22].

### 2.3 Laboratory Analyses

Metabolic and routine laboratory assessments were carried out as described previously [18]. Whole‐body insulin sensitivity was assessed by means of the hyperinsulinemic‐euglycaemic clamp, and is reported as the *M* value, which is calculated from the mean glucose infusion rates with glucose space correction during the steady‐state period [18]. All participants without diabetes underwent a 75‐g oral glucose tolerance test (OGTT) at baseline and 5‐year follow‐up to exclude dysglycaemia.

### 2.4 Metabolic Syndrome

International Diabetes Federation (IDF) 2006 criteria were used to define metabolic syndrome [23], where central obesity (waist circumference ≥ 94 cm for men and ≥ 80 cm for women in the Caucasian population) is mandatory for the diagnosis of metabolic syndrome, followed by any two of the other four components: (i) elevated triglycerides ≥ 1.7 mmol/L (150 mg/dL) or specific treatment for hypertriglyceridaemia (MetS‐TRG), (ii) reduced HDL cholesterol < 1.03 mmol/L (40 mg/dL) in men, < 1.29 mmol/L (50 mg/dL) in women or specific treatment for dyslipidaemia (MetS‐HDL), (iii) elevated blood pressure defined as systolic blood pressure ≥ 130 or diastolic blood pressure ≥ 85 mmHg or treatment of previously diagnosed arterial hypertension (MetS‐AH), (iv) impaired fasting glucose (IFG) ≥ 5.6 mmol/L (100 mg/dL) or previously diagnosed type 2 diabetes.

### 2.5 Anthropometric Assessment

Anthropometric body measurements included height, weight, waist and hip circumferences, which were measured by trained personnel using standard protocols and derived ratios [BMI and waist‐to‐hip ratio (WHR)]. Body weight was measured to the nearest 0.1 kg in light clothing. Waist circumference was measured at the midpoint between the lower edge of the rib cage and the iliac crest, and hip circumference was measured at the maximum projection of the hips, both to the nearest 0.1 cm. The cut‐off values for overweight and general obesity were estimated as a BMI ≥ 25.0 kg/m^2^ and ≥ 30.0 kg/m^2^, respectively.

### 2.6 Statistical Analysis

Data are reported as mean ± standard deviation (SD) or as percentages. Dichotomous variables were compared with the χ^2^ test and presented as the proportion of participants. Continuous variables were evaluated using the parametric *t*‐test or the nonparametric Mann‐Whitney *U* test. Correlations between the two variables were examined using Spearman's rank correlation. For multiple linear regression analyses, continuous dependent variables with skewed distributions were log‐transformed. Factors associated with dichotomous variables were examined using logistic regression analyses. Group comparisons and associations with peripheral nerve tests were adjusted for age, sex, height (except for BMI, BMI derived indices, and WHR), history of smoking, and HbA1c. Odds ratios (ORs) with corresponding 95% CIs were calculated separately in both groups for associations between categorical body composition and metabolic syndrome indices at baseline and neuropathic deficits and DSPN at follow‐up. Prospective data were analysed using Wilcoxon's test for continuous variables and McNemar's test for dichotomous variables. All statistical tests were two‐sided, with a significance threshold of *α* = 0.05. All analyses were performed using SPSS software, version 24 (IBM Corporation, Chicago, IL).

## 3 Results

### 3.1 Baseline Cohort

Baseline demographic, clinical, and neurological characteristics of participants with recently diagnosed type 2 diabetes and those with NGT are listed in Table 1. Individuals with type 2 diabetes exhibited higher body weight, BMI, waist circumference, WHR, a history of smoking, HbA1c, fasting glucose, and triglycerides, whereas HDL cholesterol and M‐value were lower compared to the NGT group. The prevalence of overweight, general and central obesity, MetS, the total number of MetS components and every single non‐glycaemic component of MetS were higher in the type 2 diabetes group compared with the NGT group. Treatment with lipid‐lowering and antihypertensive medications was more prevalent in the type 2 diabetes group compared with those with NGT. Treatment with insulin or other glucose‐lowering medications was present only in participants with type 2 diabetes. After adjustment for age, sex, height, history of smoking, and HbA1c, the ulnar, median, and peroneal MNCV, median SNCV, sural SNAP, and IENFD were lower, while WDT at the foot, NSS and NDS scores, and prevalence of DSPN were higher in the type 2 diabetes group compared with NGT. For the remaining variables, no differences were observed between the two groups.

**TABLE 1:** Demographics, clinical, and neurological characteristics of the baseline study cohort.

Variable	Normal glucose tolerance	Recent‐onset type 2 diabetes
*n* (% male)	181 (61)	355 (66)
Age (years)	44.0 ± 14.0	45.0 ± 7.7
Height (cm)	175 ± 9	174 ± 10
Weight (kg)	82.8 ± 18.5	97.8 ± 20.8^a^
BMI (kg/m^2^)	26.8 ± 5.1	32.3 ± 6.5^a^
History of smoking (%)	49	63^a^
Known diabetes duration (months)	—	5.87 ± 3.29
Waist circumference (cm)	91 ± 16	106 ± 15^a^
Waist‐to‐hip ratio	0.89 ± 0.09	0.96 ± 0.08^a^
Overweight (%)	59.1	89.0^a^
General obesity (%)	19.9	61.4^a^
HbA1c (%)	5.2 ± 0.3	6.5 ± 1.0^a^
HbA1c (mmol/mol)	33.4 ± 3.1	47.4 ± 11.4^a^
Fasting glucose (mmol/L)	4.81 ± 0.36	7.32 ± 2.41^a^
M value (mmol × min^−1^ × kg^−1^)	10.82 ± 3.58	5.98 ± 2.49^a^
Triglycerides (mmol/L)	1.30 ± 1.29	1.92 ± 1.95^a^
Cholesterol (mmol/L)	5.10 ± 1.03	5.15 ± 1.11
HDL cholesterol (mmol/L)	1.62 ± 0.46	1.18 ± 0.31^a^
LDL cholesterol (mmol/L)	3.20 ± 0.96	3.28 ± 0.97
Creatinine (μmol/L)	78.4 ± 14.0	77.0 ± 15.1
Systolic blood pressure (mmHg)	125 ± 17	124 ± 18
Diastolic blood pressure (mmHg)	70 ± 11	71 ± 11
Metabolic syndrome (%)	16	83^a^
Central obesity (%)	54.1	85.6^a^
Reduced HDL cholesterol (%)	12.4	52.7^a^
Raised triglycerides (%)	17.5	40.0^a^
Arterial hypertension (%)	56.7	86.0^a^
Number of metabolic syndrome components	1.40 ± 1.14	3.62 ± 1.01^a^
Non‐insulin glucose lowering treatment (%)	0	12^a^
Insulin treatment (%)	0	64^a^
Lipid lowering treatment (%)	2	15^a^
Antihypertensive treatment (%)	8	34^a^
Median MNCV (m/s)^b^	56.2 ± 5.5	53.8 ± 4.7^a^
Ulnar MNCV (m/s)^b^	57.7 ± 3.9	56.5 ± 5.0^a^
Peroneal MNCV (m/s)^b^	46.7 ± 3.5	45.2 ± 4.7^a^
Median SNCV (m/s)^b^	55.0 ± 6.7	52.6 ± 7.4^a^
Ulnar SNCV (m/s)^b^	53.9 ± 6.7	54.2 ± 6.6
Sural SNCV (m/s)^b^	45.8 ± 4.9	45.0 ± 5.7
Sural SNAP (μV)^b^	11.65 ± 5.28	9.96 ± 6.27^a^
Thenar CDT (°C)^b^	30.5 ± 0.8	30.3 ± 1.1
Thenar WDT (°C)^b^	33.8 ± 1.1	34.1 ± 1.5
Foot CDT (°C)^b^	28.5 ± 2.8	28.0 ± 3.6
Foot WDT (°C)^b^	38.4 ± 3.5	39.5 ± 3.8^a^
Metacarpal VPT (μm)^b^	0.41 ± 0.32	0.43 ± 0.03
Malleolar VPT (μm)^b^	1.15 ± 1.75	1.22 ± 1.38
IENFD^b^ ^,^ ^c^	10.39 ± 3.47	7.24 ± 3.62^a^
NSS (points)^b^	0.31 ± 1.33 (5.0)	1.03 ± 2.29^a^
NDS (points)^b^	0.52 ± 1.14 (3.9)	1.12 ± 1.73^a^
DSPN (%)^b^	8.9	29.9^a^

*Note:* Data are expressed as percentages or mean ± SD.

Abbreviations: CDT, cold detection threshold; DSPN, distal sensorimotor polyneuropathy; IENFD, intraepidermal nerve fibre density; MNCV, motor nerve conduction velocity; NDS, neuropathy disability score; NSS, neuropathy symptoms score; SNAP, sensory nerve action potential; SNCV, sensory nerve conduction velocity; VPT, vibration perception threshold; WDT, warmth detection threshold.

^a^ indicates *p* < 0.05 versus NGT.

^b^ indicates variables for which adjustment for age, sex, height, history of smoking, and HbA1c was performed.

^c^ *n* = 117/100.

#### 3.1.1 Baseline Associations

**FIGURE 1:**
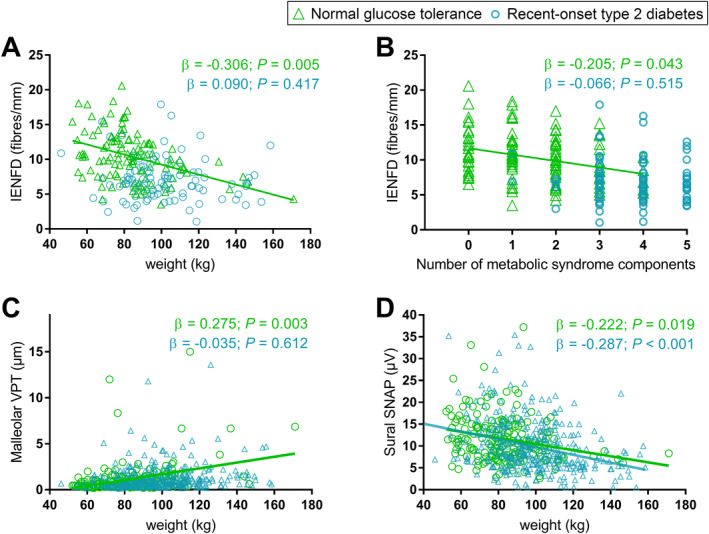
Associations of peripheral nerve tests in the lower extremity with weight and number of prevalent metabolic syndrome components at baseline. Associations of IENFD with weight (A) and the number of metabolic syndrome components (B); associations of malleolar VPT (C) and sural SNAP (D) with weight in participants with normal glucose tolerance (green) and recent‐onset type 2 diabetes (blue) at baseline. Spearman's rank coefficient and linear regression analyses adjusted for age, sex, height, history of smoking, and HbA1c. IENFD, intraepidermal nerve fibre density; SNAP, sensory nerve action potential; VPT, vibration perception threshold.

Associations between peripheral nerve tests in the lower extremity and weight or number of metabolic syndrome components that were observed after adjustment are shown in Figure 1. An inverse association was observed between sural SNAP and weight in both groups, whereas a positive association between malleolar VPT and an inverse association between weight and IENFD was only observed in the NGT group. A higher number of metabolic syndrome components was associated with a lower IENFD in the NGT group, whereas no such association was observed in the type 2 diabetes group.

In the NGT group, IENFD was also inversely associated with waist circumference (*β* = −0.311; *p* = 0.005), BMI (*β* = −0.254; *p* = 0.011), WHR (*β* = −0.254; *p* = 0.044), general obesity (*β* = −0.277; *p* = 0.003), and central obesity (*β* = −0.292; *p* = 0.007). Sural SNAP was further inversely associated with BMI (NGT/type 2 diabetes: *β* = −0.184; *p* = 0.021/*β* = −0.287; *p* < 0.001) and waist circumference (*β* = −0.222; *p* = 0.016/*β* = −0.226; *p* < 0.001) in both groups. Inverse associations with overweight (*β* = −0.129; *p* = 0.030), general obesity (*β* = −0.290; *p* < 0.001), central obesity (*β* = −0.165; *p* = 0.005), and metabolic syndrome (*β* = −0.146; *p* = 0.014) were observed only in the type 2 diabetes group. In addition to weight, malleolar VPT was also associated with waist circumference (*β* = 0.258; *p* = 0.004) in the NGT group, while an association with higher BMI was observed in both groups (*β* = 0.221; *p* = 0.005/*β* = 0.127; *p* = 0.042). In the type 2 diabetes group, higher weight, BMI, and waist circumference were associated with higher NDS (*β* = 0.197; *p* = 0.003/*β* = 0.206; *p* = 0.001/*β* = 0.151; *p* = 0.013) and with DSPN (*β* = 0.214; *p* = 0.001/*β* = 0.205; *p* = 0.001/*β* = 0.171; *p* = 0.005), while only higher weight was associated with an abnormal NDS (*β* = 0.159; *p* = 0.018).

Further associations of the entire baseline cohort of peripheral nerve function tests, including both upper and lower extremities, IENFD, and clinical neuropathy scores, for which at least two associations with anthropometric or metabolic syndrome indices were observed after adjustment, are given in Supporting Information S1: Table S1.

### 3.2 Prospective Cohort

Characteristics of the prospective cohort are provided in Table 2. No changes in glycaemic status were observed between baseline and follow‐up; no participant progressed from NGT to prediabetic stages or type 2 diabetes. Group differences in the prospective cohort at baseline were largely comparable to the entire baseline cohort. In contrast, no differences were observed in history of smoking, median SNCV, sural SNAP, and foot WDT, whereas sural SNCV was lower in the prospective type 2 diabetes group at baseline compared with NGT.

**TABLE 2:** Demographics, clinical, and neurological characteristics of the prospective study cohort.

Variable	Normal glucose tolerance		Type 2 diabetes	
	Baseline	5‐year follow‐up	Baseline	5‐year follow‐up
*n* (% male)	64 (61)	64 (61)	198 (66)	198 (66)
Age (years)	48.7 ± 13.1	53.9 ± 13.1^a^	45.7 ± 6.8	50.8 ± 6.8^a^
Height (cm)	176 ± 10	176 ± 10	175 ± 9	174 ± 9
Weight (kg)	88.0 ± 21.0	89.6 ± 21.2^a^	97.9 ± 21.6^b^	97.8 ± 20.7^b^
BMI (kg/m^2^)	28.3 ± 5.7	28.9 ± 5.8^a^	32.1 ± 6.6^b^	32.1 ± 6.2^b^
History of smoking (%)	50	50	58	62
Known diabetes duration (months)	—	—	5.93 ± 3.30	67.9 ± 4.16^a^
Waist circumference (cm)	96.9 ± 16.8	99 ± 17^a^	106 ± 16^b^	107 ± 15^b^
Waist‐to‐Hip ratio	0.92 ± 0.08	0.92 ± 0.08	0.96 ± 0.08^b^	0.96 ± 0.08^b^
Overweight (%)	75.0	76.6	88.4^b^	89.4^b^
General obesity (%)	29.7	35.9	60.1^b^	60.1^b^
HbA1c (%)	5.26 ± 0.26	5.32 ± 0.35	6.41 ± 0.86^b^	7.13 ± 1.22^a^ ^,^ ^b^
HbA1c (mmol/mol)	34.0 ± 2.8	34.6 ± 3.8	46.6 ± 9.3^b^	54.4 ± 13.3^a^ ^,^ ^b^
Fasting glucose (mmol/L)	4.84 ± 0.38	4.94 ± 0.36	7.32 ± 2.82^b^	8.89 ± 2.70^a^ ^,^ ^b^
M value (mmol × min^−1^ × kg^−1^)	10.35 ± 3.68	9.63 ± 3.40	6.43 ± 2.59^b^	5.63 ± 2.51^a^ ^,^ ^b^
Triglycerides (mmol/L)	1.25 ± 0.63	1.25 ± 0.65	1.96 ± 2.27^b^	2.30 ± 1.62^b^
Cholesterol (mmol/L)	5.29 ± 0.92	5.12 ± 0.86^a^	5.17 ± 1.13	5,36 ± 1.12
HDL cholesterol (mmol/L)	1.53 ± 0.39	1.50 ± 0.35	1.18 ± 0.33^b^	1.20 ± 0.34^b^
LDL cholesterol (mmol/L)	3.43 ± 0.87	3.32 ± 0.80^a^	3.25 ± 0.94	3.48 ± 0.99
Creatinine (μmol/L)	78.9 ± 11.7	79.5 ± 13.6	77.4 ± 14.3	75.3 ± 16.3^a^
Systolic blood pressure (mmHg)	127 ± 17	125 ± 18	122 ± 18	127 ± 22^a^
Diastolic blood pressure (mmHg)	72 ± 11	70 ± 10	73 ± 10	73 ± 13
Metabolic syndrome (%)	23.4	18.8	82.2^b^	83.8^b^
Central obesity (%)	64.1	71.9	84.2^b^	89.9^a^ ^,^ ^b^
Reduced HDL cholesterol (%)	14.1	16.7	52.9^b^	54.6^b^
Raised triglycerides (%)	20.3	17.0	40.3^b^	51.6^a^ ^,^ ^b^
Arterial hypertension (%)	68.8	73.4	84.7^b^	86.4^b^
Number of metabolic syndrome components (%)	1.67 ± 1.13	1.70 ± 0.94	3.61 ± 1.01^b^	3.75 ± 1.11^a^ ^,^ ^b^
Non‐insulin glucose lowering treatment (%)	0	0	9^b^	18^a^ ^,^ ^b^
Insulin treatment (%)	0	0	60^b^	72^a^ ^,^ ^b^
Lipid lowering treatment (%)	2	7	14^b^	22^a^ ^,^ ^b^
Antihypertensive treatment (%)	11	23^a^	35^b^	49^a^ ^,^ ^b^
Median MNCV (m/s)^c^	56.2 ± 3.4	55.4 ± 3.5	54.0 ± 4.6^b^ ^,^ ^d^	53.4 ± 5.7^b^ ^,^ ^d^
Ulnar MNCV (m/s)^c^	58.1 ± 4.3	57.8 ± 5.1	56.6 ± 4.9^b^ ^,^ ^d^	55.7 ± 5.6^a^ ^,^ ^b^ ^,^ ^d^
Peroneal MNCV (m/s)^c^	46.8 ± 3.6	45.5 ± 5.3	45.3 ± 4.7^b^ ^,^ ^d^	43.7 ± 4.0^a^ ^,^ ^b^ ^,^ ^d^
Median SNCV (m/s)^c^	54.7 ± 6.0	50.8 ± 10.4^a^	53.1 ± 7.3	51.5 ± 7.7^a^
Ulnar SNCV (m/s)^c^	53.1 ± 7.1	50.5 ± 9.9	54.6 ± 6.3	52.2 ± 6.8^a^
Sural SNCV (m/s)^c^	46.9 ± 5.2	44.6 ± 8.2	45.0 ± 5.4^b^ ^,^ ^d^	44.5 ± 6.7^a^
Sural SNAP (μV)^c^	10.7 ± 4.4	10.0 ± 7.6	9.67 ± 5.28	8.75 ± 4.72^a^
Thenar CDT (°C)^c^	30.5 ± 0.9	30.2 ± 0.9	30.4 ± 1.1	30.4 ± 0.8
Thenar WDT (°C)^c^	33.8 ± 0.9	34.3 ± 1.6	33.9 ± 0.86	34.1 ± 1.1
Foot CDT (°C)^c^	28.5 ± 3.0	27.6 ± 2.3	28.2 ± 2.87	27.8 ± 3.1
Foot WDT (°C)^c^	39.1 ± 3.4	39.8 ± 3.3	39.5 ± 3.6	39.8 ± 3.7
Metacarpal VPT (μm)^c^	0.46 ± 0.37	0.59 ± 0.37^a^	0.42 ± 0.34	0.63 ± 0.48^a^
Malleolar VPT (μm)^c^	1.60 ± 2.53	1.95 ± 3.24^a^	1.24 ± 1.32	2.16 ± 2.53^a^ ^,^ ^b^ ^,^ ^d^
IENFD (fibres/mm)^c^ ^,^ ^e^	10.46 ± 3.20	10.1 ± 4.4^a^	7.49 ± 4.05^b^ ^,^ ^d^	5.81 ± 3.16^a^ ^,^ ^b^ ^,^ ^d^
NSS (points)^c^	0.11 ± 0.67	0.20 ± 1.09	0.95 ± 2.21^b^ ^,^ ^d^	1.39 ± 2.73^b^ ^,^ ^d^
NDS (points)^c^	0.63 ± 1.42	0.97 ± 1.89^a^	1.12 ± 1.63^b^ ^,^ ^d^	1.49 ± 1.88^a^
DSPN (%)^c^	4.7	12.5	27.3^b^ ^,^ ^d^	25.8^b^ ^,^ ^d^

*Note:* Data are expressed as percentages or mean ± SD.

Abbreviations: CDT, cold detection threshold; DSPN, distal sensorimotor polyneuropathy; IENFD, intraepidermal nerve fibre density; MNCV, motor nerve conduction velocity; NDS, neuropathy disability score; NSS, neuropathy symptoms score; SNAP, sensory nerve action potential; SNCV, sensory nerve conduction velocity; VPT, vibration perception threshold; WDT, warmth detection threshold.

^a^ *p* < 0.05 versus baseline.

^b^ *p* < 0.05 versus NGT.

^c^ variables adjusted for age, sex, height, history of smoking, and HbA1c.

^d^ *p* < 0.05 versus NGT after adjustment.

^e^ *n* = 48/38/67/57.

At 5‐year follow‐up, age, diabetes duration, HbA1c, fasting glucose, and systolic blood pressure were higher, whereas *M* value and creatinine levels were lower compared to baseline in the type 2 diabetes group. The prevalence of central obesity, MetS‐TRG, total number of metabolic syndrome components, and treatment with non‐insulin and insulin glucose lowering medication, antihypertensive, and lipid lowering medication had increased at follow‐up. Higher NDS and metacarpal and malleolar VPTs were observed at follow‐up in the type 2 diabetes group, whereas ulnar and peroneal MNCV, median, ulnar, and sural SNCV, sural SNAP, and IENFD were lower at follow‐up.

In the NGT group, age, weight, BMI, waist circumference, and antihypertensive treatment rate were higher at follow‐up, whereas total and LDL cholesterol levels were lower compared with baseline. Median SNCV and IENFD decreased, whereas metacarpal and malleolar VPT and NDS scores increased over the follow‐up period. At 5‐year follow‐up, median, ulnar, and peroneal MNCV and IENFD were lower, whereas malleolar VPT, NSS, and DSPN prevalence were higher in the type 2 diabetes group compared with NGT.

#### 3.2.1 Prospective Associations

Associations between weight‐related anthropometric indices and non‐glycaemic metabolic syndrome components at baseline and changes in peripheral nerve indices over the follow‐up period are shown in Table 3. After adjustment, higher weight, BMI, waist circumference, and the presence of general obesity at baseline were associated with increasing NDS over 5 years as well as with incident abnormal NDS in the NGT group. In addition, higher weight, BMI, and waist circumference were predictive of incident DSPN in NGT. In the type 2 diabetes group, logistic regression analyses for abnormal NDS at follow‐up revealed an OR of 3.00 (1.06–8.51; *p* = 0.039) and an OR of 8.25 (1.02–66.9; *p* = 0.048) for general obesity and metabolic syndrome, respectively, present at baseline, whereas ORs in the NGT group and for other metabolic syndrome components did not reach statistical significance (*p* > 0.05). Higher weight and waist circumference at baseline were associated with increasing WDT at the foot, whereas baseline MetS‐AH was associated with decreasing WDT at the hand in type 2 diabetes. The presence of MetS‐TRG at baseline was associated with declining IENFD in participants with NGT, whereas in type 2 diabetes, only MetS‐HDL at baseline was associated with increasing IENFD and WDT at the foot.

**TABLE 3:** Prospective associations between anthropometric indices and metabolic syndrome components at baseline and changes in peripheral nerve indices over 5 years in participants with normal glucose tolerance and recent‐onset type 2 diabetes.

	Group	Weight (kg)		BMI (kg/m^2^)		Waist circumference (cm)		General obesity		MetS‐HDL		MetS‐TRG		MetS‐AH	
		*β*	*p*	*β*	*p*	*β*	*p*	*β*	*p*	*Β*	*p*	*β*	*p*	*β*	*p*
ΔWDT hand (°C)	NGT	−0.019	0.920	0.033	0.832	0.069	0.688	0.013	0.928	−0.100	0.485	0.054	0.716	0.290	0.051
	T2D	−0.021	0.840	−0.017	0.842	0.029	0.756	−0.133	0.120	−0.115	0.166	−0.103	0.222	**−0.230**	0.006*
ΔWDT foot (°C)	NGT	0.089	0.668	0.090	0.602	0.206	0.279	0.202	0.211	−0.197	0.213	0.135	0.408	0.109	0.515
	T2D	**0.289**	0.005*	0.246	0.005	**0.255**	0.005*	0.170	0.049	**0.207**	0.013*	0.101	0.239	0.141	0.098
ΔIENFD (fibres/mm)	NGT	0.003	0.991	0.015	0.943	−0.214	0.393	0.155	0.438	0.178	0.371	**−0.466**	0.026*	−0.131	0.541
	T2D	−0.072	0.718	−0.048	0.786	−0.159	0.384	−0.072	0.674	**0.381**	0.021*	0.063	0.727	−0.121	0.454
ΔNDS (points)	NGT	**0.362**	0.029*	**0.319**	0.023*	**0.372**	0.016*	**0.378**	0.004*	0.022	0.868	−0.105	0.447	0.161	0.238
	T2D	−0.080	0.423	−0.092	0.285	−0.056	0.529	−0.035	0.683	−0.033	0.691	−0.083	0.317	0.165	0.045
ΔAbnormal NDS	NGT	**0.456**	0.007*	**0.373**	0.009*	**0.449**	0.005*	**0.337**	0.013*	0.011	0.935	−0.256	0.071	0.141	0.317
	T2D	−0.020	0.839	−0.040	0.647	−0.007	0.941	0.035	0.683	0.008	0.927	−0.045	0.595	0.236	0.004
ΔDSPN	NGT	**0.667**	**<** 0.001*	**0.568**	**<** 0.001*	**0.607**	0.001*	0.312	0.045	−0.051	0.732	−0.325	0.033	0.084	0.598
	T2D	−0.172	0.108	−0.153	0.103	−0.122	0.204	−0.014	0.880	−0.124	0.162	−0.093	0.309	0.142	0.114

*Note:* Spearman's rank coefficient and linear regression analyses. * and boldface indicate *p* < 0.05 before and after adjustment for age, sex, height (except for BMI and BMI derived indices), history of smoking, and HbA1c.

Abbreviations: DSPN, distal sensorimotor polyneuropathy; IENFD, intraepidermal nerve fibre density; MetS‐AH, arterial hypertension or specific treatment; MetS‐HDL, reduced HDL cholesterol or specific treatment; MetS‐TRG, hypertriglyceridaemia or specific treatment; NDS, neuropathy disability score; NGT, normal glucose tolerance; T2D, recent‐onset type 2 diabetes; WDT, warmth detection threshold.

Additional associations between weight‐related anthropometric indices at baseline and continuous peripheral nerve indices at follow‐up are shown in Supporting Information S2: Table S2. In the NGT group, higher WHR and overweight present at baseline were associated with lower IENFD and both baseline overweight and central obesity were associated with lower CDT at the hand at follow‐up. Higher baseline BMI was also associated with higher metacarpal VPT at follow‐up only in the NGT group. None of these associations were observed in individuals with type 2 diabetes. After exclusion of associations present at baseline in the prospective cohort, no other associations were observed between baseline anthropometric or metabolic syndrome characteristics and DSPN measures at follow‐up (*p* > 0.05).

## 4 Discussion

The study's results reveal an independent relationship between higher weight and beginning nerve fibre loss and dysfunction, even in the absence of hyperglycaemia. In individuals with well‐controlled recent‐onset type 2 diabetes, general obesity and the presence of metabolic syndrome prevalent within the first year after diabetes diagnosis were predictive of neuropathic deficits 5 years later. These findings suggest that future strategies to prevent DSPN should increasingly focus on weight management to preserve nerve function and skin innervation in people with obesity, even before the manifestation of type 2 diabetes.

### 4.1 Cross‐Sectional Analysis

One of the novel findings of the present study is the observed independent association between weight‐related anthropometric indices and lower IENFD in individuals with NGT. In addition, the presence of more metabolic syndrome components was also associated with lower IENFD in this group, whereas no individual metabolic syndrome component other than central obesity was associated with lower IENFD. In contrast to prior studies [16, 17] aiming to explore the relationship between IENFD and obesity in people without manifest type 2 diabetes, participants in the present study were not selected by weight or obesity status, and prediabetic stages were excluded by an OGTT. Of note, the mean BMI of the NGT baseline cohort (26.8 ± 5.1 kg/m^2^) closely matched the BMI of the correspondent population (German adult population: 26.6 kg/m^2^ according to recent World Health Organisation estimates [24]). In a smaller cross‐sectional study, Callaghan et al. [16] reported lower IENFD in obese participants compared with lean control individuals with NGT. However, more than half of the individuals in the obese group had prediabetes or diabetes. A more recent study of the same research group [17] showed no associations between IENFD and glycaemic status, but the distribution of different glycaemic states across the study cohort was not provided. Similar to the inverse association between IENFD and the number of metabolic syndrome components present in our NGT group only, a recent study including a large number of participants with peripheral neuropathy of diabetic or cryptogenic aetiology reported a higher peripheral neuropathy severity with an increasing number of metabolic syndrome components in participants with NGT, but not in those with prediabetes or diabetes [25]. However, this association was not significant in the adjusted model, in contrast to associations between neuropathy severity and BMI or diabetes.

In contrast to previous assumptions that obesity‐related nerve damage may primarily affect small nerve fibres that are structurally assessed by IENFD [6, 26], associations between weight‐related anthropometric measures and peripheral nerve indices in the NGT group were also observed for large fibre indices (VPT, sural SNAP), indicating a parallel involvement of both small and large nerve fibres in association with higher weight. Sural SNAP was similarly associated with weight, waist circumference, and BMI in both participants with NGT and recent‐onset type 2 diabetes, suggesting a similar influence of body composition on sensory nerve axons irrespective of glycaemic status.

Only a few larger studies have previously analysed associations between nerve conduction studies and obesity [15, 27, 28]. Similar to our results, Smith and Singleton [27] observed a correlation between sural SNAP, but not with peroneal MNCV, and BMI in participants with type 2 diabetes. However, participants had a longer diabetes duration and analyses were not adjusted for other confounding factors such as glycaemic control. In a large cohort study, Callaghan et al. [15] reported associations between waist circumference and lower peroneal motor compound action potential and higher VPT, but not peroneal MNCV. In contrast to the present study, the cohort of elderly individuals also included those with prediabetes and type 1 diabetes. In the population‐based Rotterdam study, Hanewinckel et al. [28] reported a relationship between metabolic syndrome and sural SNAP in a cohort including elderly individuals with and without diabetes or IFG, but no analyses with continuous anthropometric indices were performed.

In the present study, weight, waist circumference, and BMI were associated with neuropathic deficits and prevalent DSPN in the type 2 diabetes group, demonstrating a role of higher weight per se (not restricted to obesity) as an independent risk factor for early DSPN in recent‐onset type 2 diabetes. Comparably, we previously reported associations between higher waist circumference and prevalent as well as incident DSPN in elderly individuals with and without diabetes from the population‐based Cooperative Health Research in the Region Augsburg (KORA) study [11, 12]. Moreover, in the population‐based Shanghai Diabetic Neuropathy Epidemiology and Molecular Genetics Study (SH‐DREAMS), higher waist circumference, rather than general obesity, was associated with an elevated risk for possible DSPN among Han Chinese individuals with different glycaemic states [13].

### 4.2 Prospective Analysis

In individuals with NGT, higher weight and other weight‐related anthropometric indices at baseline were associated with incident neuropathic deficits and DSPN, and lower nerve function and skin innervation at follow‐up. Incident neuropathic deficits and DSPN in the NGT group could not be explained by any other clinical condition or newly‐developed hyperglycaemia at the time of the clinical and neurological examination. Therefore, we cannot rule out a weight‐related DSPN in these individuals.

In recent‐onset type 2 diabetes, higher baseline weight and waist circumference, and MetS‐HDL were predictive for worsening WDT at the foot, while general obesity and metabolic syndrome at baseline were associated with a higher odds of clinically manifest neuropathic deficits at follow‐up, suggesting a potential role of weight in the development of peripheral nerve impairment in the early course of well‐controlled type 2 diabetes.

Obesity is recognised as a chronic, low‐grade inflammatory disorder that can trigger complex pathways of metabolic and inflammatory dysregulation [29]. Several recent preclinical studies have observed nerve damage in different rodent models of obesity, even without development of type 2 diabetes, and that immune cells may play a role in early obesity‐induced neuropathy [30]. In addition, it has been reported that modifying the immune cell profile of the peripheral nervous system in mice fed a western diet is associated with an improvement in sensory nerve function indices [31]. In the population‐based KORA F4/FF4 cohort, we recently reported associations between obesity and incident DSPN among elderly individuals with and without diabetes, and this association was partly mediated by systemic pro‐inflammatory markers [12]. Therefore, subclinical inflammation may play a critical role in the development of obesity‐induced nerve damage and may be addressed by immunomodulatory therapy approaches when weight loss interventions have failed [32, 33].

Two paradoxical associations were observed between the presence of MetS‐HDL and MetS‐AH at baseline and an improvement in some small nerve fibre measures (IENFD, WDT at the hand) in the type 2 diabetes group. However, these could be explained by an early initiation of antihypertensive and lipid‐lowering treatment and lifestyle modification in these individuals, as it is recommended for people diagnosed with type 2 diabetes to optimise cardiovascular risk factors by recent European Society of Cardiology (ESC) guidelines [34] and implemented in the Disease Management Programme for type 2 diabetes in Germany [35]. Consistent with this, weight and BMI increased over the follow‐up period in the NGT group but not in the type 2 diabetes group.

The results of the present study underscore the need to further explore the effect of weight loss interventions on DSPN outcomes in people with and without type 2 diabetes or prediabetes. Several, albeit largely uncontrolled, studies have demonstrated improvements in neuropathic symptoms associated with weight loss [36, 37, 38], whereas effects on objective outcomes such as IENFD and nerve conduction studies were inconclusive [6]. We are not aware of any larger study that evaluated the effects of weight‐loss drugs such as glucagon‐like peptide‐1 (GLP‐1) receptor agonists, dual GLP‐1 and glucose‐dependent insulinotropic polypeptide (GIP) agonists [6] on objective DSPN outcomes. Based on the present results, it is reasonable to assume that the development of DSPN may be delayed or even prevented by pharmaceutically induced weight loss in people with obesity. Thus, we see an urgent need to conduct such clinical trials.

Strengths of the present study include the adequate sample size of participants with NGT and recently diagnosed well‐controlled type 2 diabetes matched for age and sex, while unselected for weight or obesity, the inclusion of a prospective cohort, the comprehensive phenotyping including the gold standard methods to assess DSPN for both small and large nerve fibres, and adjustment for several potential confounders including glycaemic control in both groups. Limitations include the observational design that does not allow to draw conclusions about the causality of the findings presented. Further limitations include the small size of the prospective NGT group over 5 years, which is due to a delayed recruitment of participants with NGT in the GDS, which began approximately 8 years after the start of the GDS, as well as the ongoing nature of the study. In addition, the generalisability of the study to diverse ethnic groups may be limited due to the mainly Caucasian study population. The cohort characteristics of the GDS with average body composition in the NGT group and with well‐controlled, recent‐onset type 2 diabetes may limit the ability to observe associations between risk factors and DSPN compared with other cohorts with a higher prevalence of obesity, more participants with poorly controlled type 2 diabetes, and subgroups with prediabetes. However, this may also increase confidence in the robustness of the observed findings.

In conclusion, this study strengthens the role of higher weight as an independent risk factor for lower nerve fibre function and integrity in both people with NGT and well‐controlled, recent‐onset type 2 diabetes. It demonstrates the predictive value of general obesity and metabolic syndrome for neuropathic deficits along the course of early type 2 diabetes. Further clinical trials are warranted to explore the effects of weight loss interventions on objective neuropathy outcomes to prevent DSPN.

## Author Contributions

**Gundega Sipola:** investigation, data curation, formal analysis, visualization, writing – original draft, writing – review and editing. **Alexander Strom:** data curation, methodology, formal analysis, writing – review and editing. **Robert Wagner:** project administration, writing – review and editing. **Kálmán B. Bódis:** investigation, writing – review and editing. **Dan Ziegler:** methodology, supervision, project administration, writing – review and editing. **Michael Roden:** project administration, funding acquisition, writing – review and editing. **Gidon J. Bönhof:** investigation, data curation, conceptualization, formal analysis, visualization, writing – original draft, writing – review and editing. Gidon J. Bönhof acts as the guarantor of this work, had unrestricted access to all study data, and takes full responsibility for the integrity of the data and the accuracy of the analyses. The final manuscript was approved by all authors.

## Funding

The GDS was initiated and financed by the German Diabetes Center (DDZ), which is funded by the German Federal Ministry of Health (Berlin, Germany), the Ministry of Innovation, Science, Research and Technology of the State of North Rhine‐Westphalia (Düsseldorf, Germany) and grants from the German Federal Ministry of Education and Research (BMBF) to the German Center for Diabetes Research e.V. (DZD).

## Conflicts of Interest

The authors declare no conflicts of interest.

## Supporting information


Supporting Information S1

Supporting Information S2

## Data Availability

Due to ethical considerations regarding participant confidentiality and intellectual property, the datasets generated and/or analysed in this study are not publicly available. Access may be granted by the corresponding author upon reasonable request.
