# Supplementary material for: Obesity and Metabolic Syndrome Predict Polyneuropathy Over 5 Years in Recent‐Onset Type 2 Diabetes and Normal Glucose Tolerance
Source: Diabetes Metab Res Rev. 2026 Mar 3;42(3):e70147. doi: 10.1002/dmrr.70147 (PMC12956040; doi:10.1002/dmrr.70147)
Supplement: Supplementary file 1 — Supporting Information S1 [file DMRR-42-e70147-s002.docx]

**Supplemental Table 1: Associations of peripheral nerve indices observed for at least two anthropometric and metabolic syndrome characteristics at baseline.**

| Variable | Group | Weight (kg) | | BMI (kg/m²) | | Waist circumference (cm) | | Overweight | | General obesity | | Central obesity | | Metabolic syndrome | |
| --- | --- | --- | --- | --- | --- | --- | --- | --- | --- | --- | --- | --- | --- | --- | --- |
|  |  | β | P | β | P | β | P | β | P | β | P | β | P | β | P |
| Median MNCV (m/s) | NGT | -0.084 | 0.383 | -0.052 | 0.535 | -0.053 | 0.571 | -0.064 | 0.478 | -0.039 | 0.613 | 0.023 | 0.788 | 0.008 | 0.911 |
|  | T2D | **-0.182** | **0.007*** | **-0.147** | **0.016*** | **-0.142** | **0.022*** | **-0.159** | **0.007*** | **-0.204** | **0.001*** | **-0.163** | **0.007*** | **-0.15** | **0.014*** |
| Sural SNAP (µV) | NGT | **-0.222** | **0.019*** | **-0.184** | **0.021*** | **-0.222** | **0.016*** | -0.109 | 0.210 | -0.095 | 0.202 | -0.087 | 0.324 | -0.011 | 0.883 |
|  | T2D | **-0.287** | **<0.001*** | **-0.287** | **<0.001*** | **-0.226** | **<0.001*** | **-0.129** | **0.030*** | **-0.290** | **<0.001*** | **-0.165** | **0.005*** | **-0.146** | **0.014*** |
| WDT hand (ºC) | NGT | 0.123 | 0.249 | 0.094 | 0.287 | 0.063 | 0.542 | 0.013 | 0.891 | 0.076 | 0.357 | 0.054 | 0.582 | -0.030 | 0.724 |
|  | T2D | 0.085 | 0.222 | 0.078 | 0.215 | 0.070 | 0.274 | **0.130** | **0.033*** | 0.086 | 0.167 | **0.126** | **0.045*** | 0.111 | 0.075 |
| Metacarpal VPT (µm) | NGT | **0.219** | **0.018*** | **0.174** | **0.028*** | **0.24** | **0.007*** | -0.003 | 0.976 | **0.165** | **0.024*** | 0.052 | 0.546 | 0.038 | 0.611 |
|  | T2D | -0.035 | 0.612 | -0.023 | 0.714 | -0.027 | 0.673 | 0.035 | 0.562 | -0.040 | 0.517 | -0.019 | 0.756 | -0.016 | 0.796 |
| Malleolar VPT (µm) | NGT | **0.275** | **0.003*** | **0.221** | **0.005*** | **0.258** | **0.004*** | -0.035 | 0.678 | 0.098 | 0.182 | 0.065 | 0.447 | 0.020 | 0.795 |
|  | T2D | 0.107 | 0.110 | **0.127** | **0.042*** | 0.095 | 0.123 | -0.023 | 0.700 | 0.095 | 0.123 | 0.039 | 0.519 | 0.021 | 0.726 |
| IENFD (fibres/mm) | NGT | **-0.306** | **0.005*** | **-0.254** | **0.011*** | **-0.311** | **0.005*** | -0.156 | 0.165 | **-0.277** | **0.003*** | **-0.292** | **0.007*** | -0.088 | 0.365 |
|  | T2D | 0.09 | 0.417 | 0.067 | 0.534 | 0.087 | 0.402 | 0.069 | 0.503 | 0.023 | 0.826 | 0.086 | 0.401 | 0.128 | 0.206 |
| NDS (points) | NGT | -0.078 | 0.375 | -0.082 | 0.290 | -0.047 | 0.583 | -0.017 | 0.840 | -0.139 | 0.055 | 0.011 | 0.889 | 0.021 | 0.762 |
|  | T2D | **0.197** | **0.003*** | **0.206** | **0.001*** | **0.151** | **0.013*** | 0.086 | 0.146 | 0.143 | 0.017 | 0.125 | 0.036 | 0.095 | 0.112 |

Spearman’s rank coefficient and linear regression analyses. * and boldface indicate P < 0.05 before and after adjustment for age, sex, height (except for BMI and BMI derived indices), history of smoking, and HbA1c.

MNCV, motor nerve conduction velocity; NGT, normal glucose tolerance; T2D, recent-onset type 2 diabetes; SNAP, sensory nerve action potential; WDT, warmth detection threshold; VPT, vibration perception threshold; IENFD, intraepidermal nerve fibre density; NDS, Neuropathy Disability Score.
