# Supplementary material for: Obesity and Metabolic Syndrome Predict Polyneuropathy Over 5 Years in Recent‐Onset Type 2 Diabetes and Normal Glucose Tolerance
Source: Diabetes Metab Res Rev. 2026 Mar 3;42(3):e70147. doi: 10.1002/dmrr.70147 (PMC12956040; doi:10.1002/dmrr.70147)
Supplement: Supplementary file 2 — Supporting Information S2 [file DMRR-42-e70147-s001.docx]

**Supplemental table 2: Associations between weight-related anthropometric indices at baseline and peripheral nerve indices at 5-year follow-up in participants with normal glucose tolerance and recent-onset type 2 diabetes.**

| Baseline  variable  Follow-up  variable | Group | Weight (kg) | | BMI (kg/m²) | | Overweight | | Waist circumference (cm) | | WHR | | Central obesity | |
| --- | --- | --- | --- | --- | --- | --- | --- | --- | --- | --- | --- | --- | --- |
|  |  | β | P | β | P | β | P | β | P | β | P | β | P |
| CDT hand (ºC) | NGT | 0.015 | 0.929 | 0.021 | 0.886 | **-0.303** | **0.028*** | -0.045 | 0.775 | -0.011 | 0.943 | **-0.338** | **0.037*** |
|  | T2D | 0.083 | 0.413 | 0.092 | 0.288 | 0.102 | 0.228 | 0.077 | 0.394 | 0.078 | 0.487 | 0.112 | 0.198 |
| Metacarpal VPT (µm) | NGT | 0.498 | 0.001 | **0.405** | **0.002*** | 0.126 | 0.346 | 0.336 | 0.019 | -0.036 | 0.808 | 0.212 | 0.166 |
|  | T2D | 0.051 | 0.590 | 0.026 | 0.757 | 0.018 | 0.825 | 0.099 | 0.251 | 0.100 | 0.339 | 0.090 | 0.277 |
| IENFD (fibres/mm) | NGT | -0.445 | 0.030 | -0.388 | 0.021 | **-0.465** | **0.008*** | -0.570 | 0.004 | **-0.403** | **0.033*** | -0.450 | 0.054 |
|  | T2D | 0.004 | 0.980 | -0.014 | 0.918 | 0.109 | 0.375 | -0.065 | 0.635 | -0.082 | 0.601 | 0.022 | 0.865 |

Spearman’s rank coefficient and linear regression analyses. * and boldface indicate P < 0.05 before and after adjustment for age, sex, height (except for BMI, overweight, and WHR), history of smoking, and HbA1c and P > 0.05 with respective peripheral nerve index at baseline.

WHR, waist-to-hip ratio; CDT, cold detection threshold; NGT, normal glucose tolerance; T2D, recent-onset type 2 diabetes; VPT, vibration perception threshold; IENFD, intraepidermal nerve fibre density.
